# *FASN*, *SCD*, and *PLAG1* Gene Polymorphism and Association with Carcass Traits and Fatty Acid Profile in Hanwoo Cattle

**DOI:** 10.3390/ani15060897

**Published:** 2025-03-20

**Authors:** Jia Yu, Sajida Naseem, Sungkwon Park, Sunjin Hur, Yoonbin Choi, Teahyung Lee, Xiangzi Li, Seongho Choi

**Affiliations:** 1 Department of Animal Science, Chungbuk National University, Cheongju 28644, Republic of Korea; sophiayu789@gmail.com (J.Y.); yb3232@chungbuk.ac.kr (Y.C.); jijone@dodram.co.kr (T.L.); 2 Engineering Research Center of North-East Cold Region Beef Cattle Science & Technology Innovation Ministry of Education, Department of Animal Science, Yanbian University, Yanji 133002, China; sajidanaseem1992@gmail.com; 3 Department of Food Science and Biotechnology, Sejong University, Seoul 05006, Republic of Korea; sungkwonpark@sejong.ac.kr; 4 Department of Animal Science and Technology, Chung-Ang University, Anseong 17546, Republic of Korea; hursj@cau.ac.kr

**Keywords:** SNPs, carcass traits, meat quality, Hanwoo

## Abstract

Genetic polymorphisms have a great impact on enhancing quantitative traits in cattle. In this study, Fatty acid synthase (*FASN*) g. 16024 (A>G), Stearoyl-CoA desaturase (*SCD*) g. 10329 (C>T), and pleomorphic adenoma gene (*PLAG1*) g. 25003338 (C>G) genotypic and allelic polymorphisms were evaluated, along with their associations with fatty acid composition, adipogenic gene expression, and carcass characteristics (carcass weight, yield grade, backfat thickness, and marbling score) in Hanwoo steers. A total of 128 Hanwoo steers were selected for this study and the Polymerase chain reaction–restriction fragment length polymorphism (PCR-RFLP) method was used to identify polymorphism of these genes. The AG genotype and G allele in *FASN* g. 16024 (A>G), CT genotype and T allele in *SCD* g. 10329 (C>T), and GG genotype and G allele in *PLAG1* g. 25003338 (C>G) showed higher frequency and positively correlated with carcass traits, yield, and quality grades. Fatty acid composition results indicate that C18:3n-6, C20:1, and C20:2n-6 were significantly higher in the AA genotype of *FASN* gene, C14:1 and C18:3n-6 in the CC genotype, and C16:1 in the TT genotype of *SCD* gene. C12:0, C14:0, C16:1, C18:0, and C20:0 were higher in the CC genotype of *PLAG1* gene. Furthermore, RT-qPCR analysis of adipogenesis-related genes (AMP-activated protein kinase-α (*AMPKα*), Carnitine palmitoyl transferase-1β (*CPT1*), G-coupled protein receptor-43 (*GPR43*), and *SCD*) across different SNP genotypes suggests a systemic interaction between genetic factors and adipogenesis in beef cattle. This study emphasizes the significance of *FASN* g. 16024 (A>G), *SCD* g. 10329 (C>T), and *PLAG1* g. 25003338 (C>G) SNPs for genetic selection to enhance beef quality and elucidate lipid metabolic pathways in Hanwoo cattle.

## Simple Summary

This study was designed to explore the single nucleotide polymorphisms (SNPs) of Stearoyl-CoA desaturase (*SCD*), Fatty acid synthase (*FASN*), and pleomorphic adenoma gene 1 (*PLAG1*) genes and their relation to meat quality, carcass weight, marbling score, yield grade, backfat thickness, and fatty acid composition in Hanwoo steers. The results showed that G allele, T allele, and G allele frequency was higher in *FASN*, *SCD*, and *PLAG1* genes, respectively. Statistical analysis revealed that the AA genotype of *FASN* gene, the TT genotype of *SCD* gene, and the CC genotype of *PLAG1* gene were significantly associated with fatty acid composition in meat and adipose tissue. Genotypes of *PLAG1* variably related to carcass weight, marbling score, yield grade, and backfat thickness. Our results suggest that these SNPs are effective for marker-assisted breeding for the improvement of meat quality and fatty acid composition in Hanwoo cattle.

## 1. Introduction

Quality grades of meat products in the commercial beef industry have significant importance. The color of meat, fat firmness, tenderness, and juiciness are important attributes consumers consider [1,2]. In Korean society, consumers generally prefer high-quality Hanwoo beef, especially for home dining and social gatherings such as hosting friends and family. This preference has contributed to a food culture that emphasizes premium ingredients. As a result, there is a strong demand for high-grade beef, particularly for traditional dishes like Korean barbecue. The market price of 1++ grade Hanwoo beef is approximately 15,500 KRW per 100 g, with higher restaurant prices due to service costs and demand. This reflects the premium on high-quality beef, where consumer willingness to pay is shaped by perceived quality and availability. Such economic factors highlight the importance of genetic selection for improving beef quality.

Numerous SNPs were found and linked to quantitative features when genome sequencing in domestic animals became available [3]. Researchers can utilize newly discovered genetic correlations to create more effective plans to increase or prevent desired characteristics in the target population [4]. Moreover, as consumers become increasingly aware of the relationship between diet and health, the fatty acid composition of meat has gained attention. SNPs in lipid metabolism-related genes, such as *FASN*, *SCD*, and *PLAG1*, have been associated with variations in fatty acid profiles in cattle [5,6]. These genetic variations influence key processes like fatty acid synthesis and desaturation, which can affect meat quality traits such as marbling and unsaturated fatty acid content [4,7]. However, the extent to which SNP variations alone can significantly modulate fatty acid composition within the same species remains a topic of discussion. While some studies suggest a strong genetic influence, others highlight the role of environmental factors, such as diet and feeding regimens, in determining fatty acid profiles [8]. Further research integrating genetic, nutritional, and management strategies is essential to fully understand the interplay between SNP variations and lipid metabolism pathways in beef cattle.

Multiple investigations have been conducted to identify significant correlations between QTLs and SNP markers to identify genotypic polymorphisms that are crucial for improving the yield of cattle [9]. Fatty acid synthase (*FASN*) was responsible for carcass traits in various cattle breeds, for marbling and fatty acid composition [10]. There are multiple SNP sites on *FASN* located on BTA 19 that are significantly associated with C14:0, C18:1 n-9, and other monounsaturated fatty acids (MUFAs) [11,12]. The *FASN* g. 16024 G>A mutation of Japanese black cattle has a significant impact on the fatty acid composition of backfat, intermuscular fat, and intramuscular fat, which is related to the ratio of C18:0 and C18:1 [13].

The *SCD* (Stearoyl-CoA desaturase) gene located on chromosome 26 is associated with more than one fatty acid trait in American Angus cattle [14]. The *SCD* g. 10329 C>T single nucleotide polymorphism in Korean steers is associated with the oleic acid, monounsaturated fatty acid content, and marbling of beef [15]. Japanese Black *SCD* genotypes showed relation with fatty acid composition and beef quality [16]. Genome-wide association analysis of Korean cattle showed that *PLAG1* (pleomorphic adenoma gene 1), located on cattle BTA 14, is related to carcass weight [17]. Previous studies have shown that *PLAG1* g. 25003338 C>G can be a functional candidate gene for trait association and genetic drift [18]. In a study on Nellore cattle, it was pointed out that *PLAG1* g. 25003338 C>G is the main pleiotropic gene for birth weight, weaning weight, and weight at one year of age. *PLAG1* may affect accessory genes involved in regulating carcass traits [19]. Recent studies indicate that *PLAG1* not only regulates growth traits but also plays a role in lipid metabolism. It influences *IGF-1* signaling, which affects adipogenesis and fatty acid metabolism [20]. Variants of *PLAG1* have been associated with subcutaneous and intramuscular fat deposition, impacting beef quality and fatty acid composition [21]. These findings suggest that *PLAG1* may modulate key genes involved in lipid regulation, further supporting its relevance in beef production.

Although *FASN* and *SCD* are known to influence fatty acid synthesis and desaturation, the specific impact of single-site polymorphisms on adipogenic gene expression remains unclear [15]. Likewise, the role of *PLAG1* in lipid metabolism beyond its function in growth regulation has not been fully explored. This study aims to bridge this gap by comprehensively examining the relationship between *FASN*, *SCD*, and *PLAG1* polymorphisms and carcass traits, adipogenic gene expression, and lipid metabolism in Hanwoo cattle. These findings will provide deeper insights into the genetic regulation of fat composition and contribute to selective breeding strategies for high-quality beef production.

## 2. Materials and Methods

### 2.1. Animals, Phenotypic Data, and Sample Collection

In this study, 128 Hanwoo cattle were used, belonging to Chungju National Agricultural Cooperative Federation, Republic of Korea. All animals used in this study were sourced from a single farm, where they were raised under uniform feeding conditions and dietary composition. The sample tissues were obtained from Farm Story Hannaeong Bio & Food Co., Ltd. (Chungbuk, Republic of Korea). For genomic DNA extraction, sampling was conducted in the pre-cooled room after the cattle were slaughtered. For extraction of genomic DNA, muscle tissue was collected from the ‘Round’ part of the carcass. The collected samples were immediately immersed in liquid nitrogen and transferred to the laboratory within 2 h. For fatty acid extraction, fat tissue was collected from the back fat. Backfat thickness, carcass weight, yield grade, and marbling score were included in carcass traits. The marbling score was numbered as 1 = trace to 9 = very abundant, according to Korean Beef Marbling Standard [22].

### 2.2. Genomic DNA Extraction and Polymerase Chain Reaction-Restriction Fragment Length Polymorphism (PCR-RFLP)

DNA was extracted from samples by using the Wizard^®^ HMW DNA Extraction Kit (Cat. # A2920, Promega, Madison, WI, USA). RFLP assay was conducted utilizing bovine muscle tissue samples to analyze the genotype.

The polymerase chain reaction (PCR) was carried out in three steps using the Solg™Taq DNA Polymerase kit (STD16-R250, SolGent Co., Ltd., Seoul, Republic of Korea). The PCR cycling conditions included an initial denaturation for 15 s at 95 °C, followed by 30 min at 95 °C, then annealing for 30 s at 52 °C for *FASN*-g.16024, 30 s at 52 °C for *SCD* g. 10329 (C>T), and 30 s at 53 °C for *PLAG1* g. 25003338 (C>G). This was succeeded by an extension for 40 s at 72 °C and a final extension for 5 min at 72 °C, over 35–40 cycles. Primer sequences are listed in Table 1. Post-PCR, a 1% agarose gel electrophoresis was used to verify DNA amplification.

Upon observing DNA bands on the electrophoresis gel, RFLP analysis was conducted to confirm DNA amplification. For RFLP, restriction enzymes from New England Biolabs (Ipswich, MA, USA) were utilized, specifically *Hha I* for *FASN* g. 16024 (A>G), *Fau I* for *SCD* g. 10329 (C>T), and *Hph I* for *PLAG1* g. 25003338 (C>G) (Table 1). The RFLP reaction mixture was thoroughly mixed and incubated overnight at 37 °C. Subsequently, electrophoresis was performed on a 3% agarose gel to analyze genotypes based on band fragmentation patterns.

### 2.3. Total RNA Extraction and RT-qPCR

Total RNA was isolated from muscle samples using the Trizol reagent by Thermo Fisher Scientific (Invitrogen, Waltham, MA, USA). The quality and quantity of the extracted RNA were assessed with a NanoDrop ND-1000 spectrophotometer (2000C, Thermo Fisher Scientific). All RNA samples displayed purity ratios (A260/A280) exceeding 1.80~2.0. For cDNA synthesis, 1 μg of total RNA was reverse-transcribed using AccuPower^®^ CycleScript™RT PreMix (dN6) from Bioneer, Daejeon, Republic of Korea.

Real-time polymerase chain reaction (RT-PCR) was then conducted using AccuPower^®^ 2× GreenStar™qPCR Master Mix (Bioneer, Daejeon, Republic of Korea) on a StepOnePlus™Real-Time PCR System from Applied Biosystems™ (Thermo Fisher Scientific, Foster City, CA, USA). RT-PCR was performed according to the protocol provided by the manufacturer. The housekeeping gene *RPS9* served as the reference. Key genes including *AMPK-α*, *CPT1*, *GPR4*, and *SCD* were selected to analyze adipogenic and lipogenic gene expression levels (Table 2). The RT-qPCR thermocycling conditions consisted of an initial preheating at 95 °C for 15 min, followed by 40 cycles of denaturation at 95 °C for 15 s and annealing at 60 °C for 1 min, concluding with a melting curve analysis ranging from 60 °C to 95 °C. Relative gene expression levels were quantified using the 2^−ΔΔCt^ method [23], enabling a thorough assessment of gene expression changes.

### 2.4. Fatty Acid Analysis

To determine the fatty acid composition, we utilized 2 g of muscle tissue and 1 g of adipose tissue, each sample mixed with Folch solution (chloroform: methanol in a 2:1 volume ratio). C13:0 was added to each sample, serving as an internal standard. After the lipids were extracted, the saponification of lipids was achieved by using 0.5 M KOH in methanol. BF3 was used for methyl esterification of fatty acids.

Gas chromatographic analysis was performed using an HP8890 system (Agilent, Santa Clara, CA, USA). The column used was an SPTM-2560 fused silica capillary column (Supelco, 100 m × 0.25 mm × 0.2 μL film thickness, Sigma-Aldrich, St. Louis, MO, USA). The injector and FID temperatures were set at 270 °C and 300 °C, respectively. The oven temperature was initially held at 150 °C for 10 min, then increased by 2 °C per minute to 220 °C, and maintained at 220 °C for a further 10 min. Column flow rate and split ratio were maintained at 10 cm/min and 100:1, respectively. Fatty acid identification was facilitated by adding a standard mixture (LRAC7954, Sigma-Aldrich, St. Louis, MO, USA), with C13:0 fatty acid (Sigma-Aldrich, St. Louis, MO, USA) used as the internal standard for precise quantification.

### 2.5. Statistical Analysis

At least three biological replicates were used for each experimental analysis. Statistical analyses were performed using SPSS 23 (IBM, Armonk, NY, USA). The chi-square test was used to assess Hardy–Weinberg equilibrium (HWE) for genotype distributions. One-way analysis of variance (ANOVA) with multiple comparisons was used to evaluate the effects of genotype on carcass traits and relative gene expression. A General Linear Model (GLM) was applied to assess the effects of genotype on fatty acid composition in muscle and adipose tissues. *p*-values from the GLM model represent the overall genotype effect. When *p* < 0.05, Duncan’s multiple range test was conducted for post-hoc pairwise comparisons. In cases where the overall genotype effect was not significant (*p* > 0.05), localized differences between specific genotype groups may still be detected due to within-group variability. Data are presented as mean ± standard error of the mean (SEM), and statistical significance was set at *p* < 0.05.

## 3. Results

### 3.1. Genotype Frequencies and Allele Frequencies with Carcass Traits in the Hanwoo Steer

#### 3.1.1. Analysis of Genotype and Allele Frequencies in Hanwoo Steers

**Figure 1:**
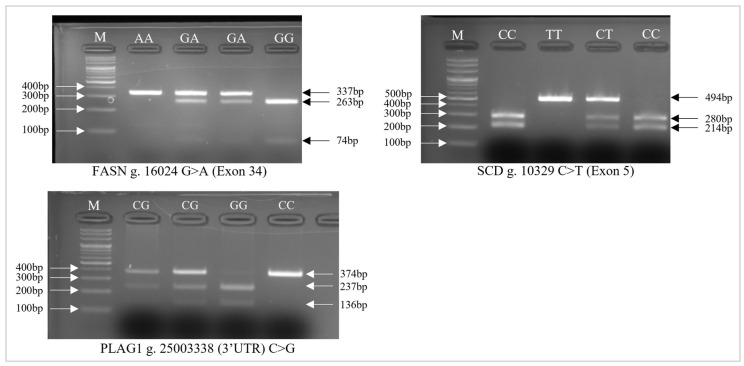
Illustration of *FASN* g. 16024 (A>G), *SCD* g. 10329 (C>T), *PLAG1* g. 25003338 (C>G) genotypes on the agarose gel. 100 bp ladder (Cat. No. SDL42-B500, SolGent™ 100 bpPlus DNA Ladder, SolGent Co., Ltd., Seoul, Republic of Korea).

The *FASN* g. 16024 (A>G), *SCD* g. 10329 (C>T), *PLAG1* g. 25003338 (C>G) polymorphism of Hanwoo detected in PCR-RFLP fragments is shown in Figure 1. The genotype and allele frequencies in Hanwoo steers were systematically compared, as detailed in Table 3. The genotype distributions were tested for Hardy–Weinberg equilibrium (HWE) using a chi-square (χ^2^) test. The results (*p* > 0.05) indicate no significant deviation from HWE, confirming that the sample population is in genetic equilibrium. Genotypic and allele frequencies showed that *FASN*-AG (41.7%) and G allele (58.8%), *SCD*-CT (54.5%) and T allele (60.6%), and *PLAG1*-GG (51.5%) and G allele (71.2%) were predominant, while homozygous AA, CC, and CC genotypes were less frequent. The higher frequency of *FASN*-AG and G allele suggests a potential selection advantage for fatty acid synthesis and marbling traits, as FASN is a key enzyme in de novo lipogenesis. The predominance of *SCD*-CT and T allele aligns with *SCD*’s role in promoting monounsaturated fatty acid (MUFA) synthesis, which is beneficial for beef tenderness and flavor. The prevalence of *PLAG1*-GG and G allele may indicate its role in growth-related pathways, potentially influencing lipid deposition and carcass traits. These findings suggest that certain genotypes may contribute to desirable beef quality traits, reinforcing their potential use as markers in selective breeding programs.

#### 3.1.2. Correlation Between Genotypes/Alleles and Beef Quality Grades

An examination of beef quality grades revealed that the 1++ grade was more frequently associated with Hanwoo steers possessing the *FASN*-AG genotype and G allele, *SCD*-CT genotype and T allele, and *PLAG1* genotype GG and G allele. Furthermore, these specific genotypes and alleles correlated with higher yield grades, indicating a positive relationship between these genotypes/alleles and superior carcass traits (Table 3). Upon aggregating marbling scores of 7, 8, and 9, genotypes and alleles associated with high marbling scores exhibited greater consistency with the observed quality grades and yield grades. These findings suggest the potential to predict the quality and yield grades of Hanwoo beef by analyzing the presence of specific alleles or genotypes.

#### 3.1.3. Differential Impact of *FASN*, *SCD*, and *PLAG1* Genotypes on Carcass Traits

**Figure 2:**
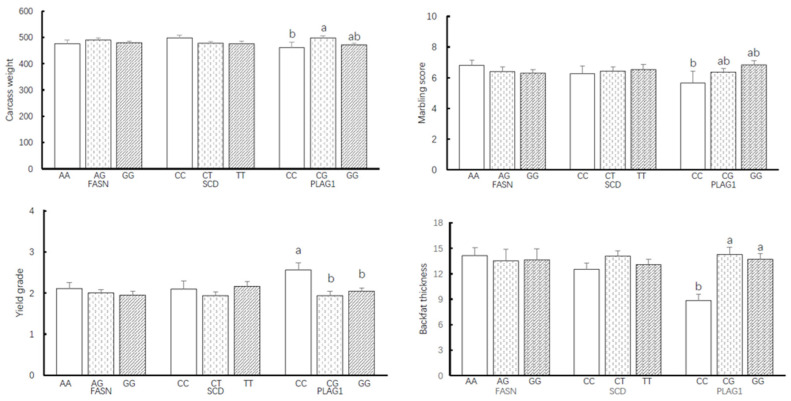
Association of carcass weight, marbling score, and yield grade with genotype of *FASN* g. 16024, *SCD* g. 10329, and *PLAG1* g. 25003338. Yield Grade A = 3, Grade B = 2, Grade C = 1. Marbling score: 1 = trace, 9 = very abundant. Yield grade: A (> 62.52), B (60.40–62.52), C (<60.40). (Yield grade thresholds are based on the livestock grading criteria issued by the Ministry of Agriculture, Food and Rural Affairs (MAFRA), Republic of Korea; Notification No. 2020-112). a, b: Mean in the same line with different superscripts significantly differed (*p* < 0.05).

Comparative analysis of carcass traits, including carcass weight, marbling score, yield grade, and backfat thickness, showed no significant differences across *FASN* and *SCD* genotypes (Figure 2). However, the analysis of the *PLAG1* genotype yielded significant findings. Hanwoo steers carrying the G allele, particularly those with CG and GG genotypes, exhibited superior results regarding carcass weight, marbling score, and backfat thickness, with statistical significance (*p* < 0.05). In contrast, the CC genotype was found to have a significantly higher sum of yield grades compared to CG and GG types, presenting an inverse relationship in terms of yield grade performance.

### 3.2. Fatty Acid Composition of Different Genotypes in Hanwoo Steers

According to Table 4, the influence of the *FASN* g. 16024 SNP is more pronounced in adipose tissue than in muscle tissue. In muscle tissue, a significant increase in unsaturated fatty acid C18:3n-6 was observed in the AA and AG genotypes compared to the GG genotype (*p* < 0.05). In adipose tissue, both saturated and unsaturated fatty acids were impacted, with higher levels of saturated fatty acid C12:0 and total saturated fatty acids in genotypes containing allele G (*p* < 0.05). Additionally, the AA genotype exhibited the highest levels of long-chain unsaturated fatty acids C20:1 (*p* < 0.05).

The impact of the *SCD* g. 10329 SNP was evident in the medium-chain unsaturated fatty acid C14:1 content, which was higher in the CC genotype and C allele (*p* < 0.05) (Table 5) in both muscle and adipose tissue. However, an inverse pattern was observed in the unsaturated to saturated fatty acids ratio and total saturated fatty acids were higher in TT genotype. Genotypes containing T allele had higher saturated fatty acid content (*p* < 0.05), but a lower ratio of unsaturated to saturated fatty acids (*p* < 0.05).

In the context of *PLAG1* genotypes, the SNP impact was more significant in adipose tissue than in muscle tissue. Medium-chain saturated fatty acids C12:0, C14:0, and total saturated fatty acids were higher in C allele containing genotypes (*p* < 0.05) in adipose tissue. Unsaturated fatty acids C18:1, C18:3, C20:1, and total monounsaturated fatty acids were elevated in genotypes containing the G allele (*p* < 0.05). On the other side, medium-chain saturated fatty acids C14:0 and monounsaturated fatty acid C16:1 were elevated in muscle tissue for C allele -containing genotypes (*p* < 0.05) (Table 6).

An analysis was conducted to determine the influence of genotype and quality grade and their interaction with fatty acid content in muscle and adipose tissue. Results indicated that for *FASN* g. 16024 and *SCD* g. 10329, the interaction between genotype and quality grade on fatty acid content was insignificant (Table 7, Table 8 and Table 9). However, there was a correlation with long-chain unsaturated fatty acid content in the case of *PLAG1* g. 25003338 C>G: the influence of genotype, quality grade, and their interactions on muscle tissue were more pronounced, affecting both medium- and long-chain saturated and unsaturated fatty acids, including C16:0, C16:1, C18:1, and C18:2, as well as total saturated and unsaturated fatty acids. The effects of the *PLAG1* g. 25003338 SNP on fatty acids are multifaceted, influencing a range of saturated and unsaturated fatty acids in muscle and adipose tissues.

### 3.3. Relative Gene Expression of Different Genotypes in Hanwoo Steer

**Figure 3:**
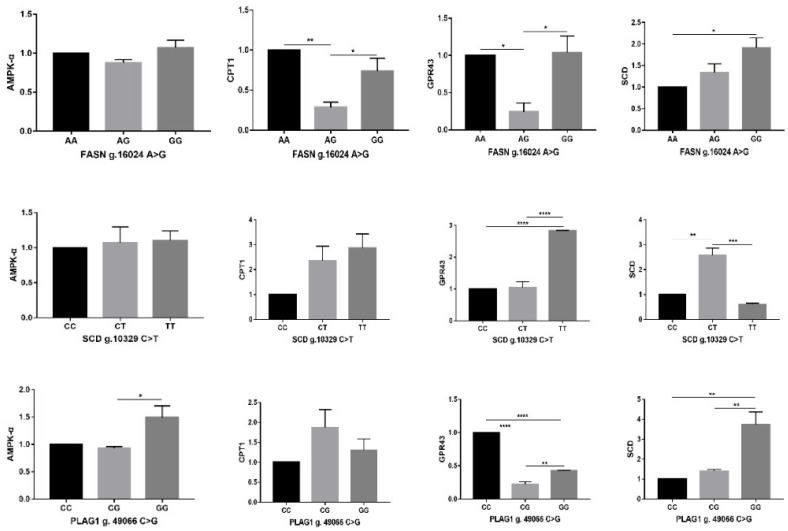
Relative gene expression of *AMPK*, *GPR43*, *CPT1*, and *SCD* in muscle tissue of Hanwoo steer. The results were expressed as means ± SEM (n = 3). The asterisks indicate a significant difference between results (* *p* < 0.05, ** *p* < 0.01, *** *p* < 0.001, **** *p* < 0.0001).

The RT-qPCR analysis was conducted to check the expression of adipogenic genes *AMPKα*, *CPT1*, *GPR43*, and *SCD*, which are integral for lipid metabolism. The analysis revealed that the expression differences of *AMPKα* among the genotypes at three polymorphic sites were not substantial. *AMPKα*, *CPT1*, and *GPR43* demonstrated similar expression patterns across the *FASN* genotypes. Specifically, the expression levels of *CPT1* and *GPR43* in *FASN*-AG were lower than in the AA and GG genotypes. Conversely, the *SCD* gene, implicated in the desaturation of fatty acids, exhibited the highest expression level in the GG genotype (*p* < 0.05) (Figure 3), while the expression levels of *AMPKα* and *CPT1* were elevated in *SCD* CT and TT genotypes; however, the differences were not statistically significant. *GPR43* showed a significantly higher expression in TT than other genotypes (*p* < 0.001). Interestingly, the highest expression of the *SCD* gene was noted in the CT genotype (*p* < 0.01), though no discernible difference was found between the CC and TT genotypes. Furthermore, the expression levels of *AMPKα* and *CPT1* were higher in *PLAG1* GG and CG genotypes, respectively, than in the CC type. The expression level of *SCD* mirrored this pattern, being higher in genotypes containing G allele; contrarily to other genes, the *GPR43* expression was higher in the *PLAG1* CC genotype (*p* < 0.01).

The study’s findings indicate a complex interaction between various genotypes and the expression levels of genes involved in adipogenic processes and lipid metabolism. This suggests a genetic influence on the metabolic pathways regulated by these genes, with potential implications for understanding lipid metabolism in different genotypes.

## 4. Discussion

Although animals were raised under standardized feeding and environmental conditions, the extended sampling period and reliance on slaughterhouse collection may introduce some variability. However, given that all animals came from the same farm with uniform feeding management, the impact of such variability is expected to be minimal.

### 4.1. Genotype Frequencies and Allele Frequencies with Carcass Traits

Quantitative traits (e.g., carcass traits, body size, yield grade, and marbling score) are controlled by multiple genes with various genetic loci, which all have a minor amount of impact on overall variations in traits. In this study, the frequency of A allele of *FASN* (g.16024G>A) (0.412) was higher than that observed in Dutch Holstein cattle (0.11) [24], and Fleckvieh bulls (0.225) [25], while lower than in Japanese Black cattle (0.85) observed by Matsuhashi [26]. The GG genotype frequency was lower in this study (0.380) as compared to Fleckvieh bulls (0.599) [25]. The CC, CT, and TT genotype frequency of *SCD* g. 10329 C>T was reported as 0.222, 0.444, and 0.333 respectively in Korean native cattle [27], which was quite variable to our study. Various breeds of cattle have been studied for *PLAG1* gene polymorphism such as Angus cattle, Hanwoo, Simmental, and Chinese cattle [21,27,28,29]. *PLAG1* g. 25003338 C>G, studied for the first time in this research, showed higher allele G frequency (0.712) than allele C; however, G allele frequency was also higher in *PLAG1* c.795A>G than A allele in Bali cattle [30]. Our work and previous studies of polymorphism for different genes suggest that genotypes and allelic frequencies have been specific to distinct cattle breeds.

Furthermore, we identified a significant correlation between specific SNP genotypes in *FASN*, *SCD*, and *PLAG1* genes and the carcass traits of Hanwoo steers. Notably, the increased frequency of *FASN*-AG and G allele, *SCD*-CT, and T allele, along with *PLAG1* GG type and G allele, indicates a genetic predisposition towards desirable carcass traits. This observation aligns with prior studies by Chu et al. [31] and Gao et al. [32], which have documented the impact of *FASN* and *SCD* genes on body traits, fat deposition, and fatty acid composition in Qinchuan cattle. Additionally, the role of the *PLAG1* gene in growth and body size regulation, as explored by Utsunomiya et al. [18] and Fink et al. [33], supports our findings.

The prevalence of beef grade 1++ in Hanwoo steers possessing these genotypes and alleles highlights the practical implications of our research. It reinforces the hypothesis that certain genotypes contribute to higher quality and yield grades in beef production. It suggests potential for utilizing genetic selection and breeding strategies to enhance beef quality in Hanwoo cattle.

Furthermore, the association between high marbling scores and the *FASN*-AG, *SCD*-CT, and *PLAG1*-GG genotypes is noteworthy, especially considering the importance of marbling in beef quality assessment. This correlation indicates that these genetic markers might be reliable predictors of marbling potential, a crucial factor in meat quality.

Regarding carcass traits, no significant variances were observed between the *FASN* and *SCD* genotypes; however, the *PLAG1* genotype showed remarkable differences. The superior performance of the CG and GG types over the CC type in carcass weight, marbling score, and backfat thickness (*p* < 0.05) is consistent with the known functions of *PLAG1* in growth regulation [34]. However, the inverse relationship in yield grade, where CC types excelled over CG and GG types, points to a more intricate genetic interplay, meriting further exploration.

In conclusion, the insights gained from this study are invaluable for genetic selection in Hanwoo cattle. Identifying genotypes correlating with higher quality and yield grades can significantly enhance breeding programs to optimize carcass traits. Employing SNP genotyping as a tool in selective breeding can transform the Hanwoo beef industry by enabling more precise genetic selection based on specific carcass characteristics.

### 4.2. Fatty Acid Composition of Different Genotypes

This study delves into the impact of SNP genotypes, specifically *FASN* g. 16024 (A>G), *SCD* g. 10329 (C>T), and *PLAG1* g. 25003338 (C>G), on the fatty acid composition of beef, uncovering several critical insights. The *FASN* and *SCD* genes, crucial for fatty acid synthesis and desaturation, respectively, strongly correlate with the beef fatty acid profile. These findings are supported by previous studies [35,36], which demonstrates a genetic predisposition affecting both saturated and unsaturated fatty acid content, thereby directly influencing the nutritional value of beef [37]. In the fatty acid results, certain differences approached significance (*p* < 0.10), suggesting potential genotype-related trends in fatty acid composition and adipogenic gene expression. While these results do not indicate conclusive statistical differences, they provide insight into possible genetic influences that warrant further investigation with larger sample sizes.

This study revealed notable correlations between specific SNP genotypes within these genes and the levels of saturated fatty acids (SFAs) and monounsaturated fatty acids (MUFAs) in beef. Bartoň [25] reported *FASN* (g.16024G>A) and *FASN* (g.17924A>G) association with atherogenic index and concentrations of C14:0, C16:0, and C18:1 n-9 fatty acids in subcutaneous and muscle fat of Fleckvieh bulls. Kim et al. [38]’s research showed a positive relation of this gene with C18:1, and negative association with muscle saturated fatty acids of Hanwoo steers, which are quite similar to this study’s results, as both saturated (C12:0) and unsaturated fatty acid in adipose and muscle tissue were affected, respectively. Previous studies on Hanwoo steers showed that *SCD* SNPs g.10329 C>T and g.14047 C>T interaction have a significant effect on C18:1 and MUFAs, and *SCD* g.10329 C>T associated with total MUFA content [39,40] in muscle tissue; contrarily, in this study, C14:1 was significantly increased by the CC genotype of *SCD* g.10329 (C>T) in muscle tissue. Variants in the *FASN* gene have been linked to increased SFA levels in adipose tissue, while certain SNPs in the *SCD* gene correlate with elevated MUFA content, this aligns with studies suggesting that genetic factors significantly impact the composition of beef fat [41]. This study emphasizes the importance of the ratio of saturated to unsaturated fatty acids in beef, a critical aspect of nutrition and health. The genotypic variations observed in the *SCD* and *FASN* genes have influence on fatty acids ratio, which has significant implications for the breeding strategies to produce beef with a healthier fatty acid profile. As an n-6 polyunsaturated fatty acid (PUFA), γC18:3 serves as a precursor for dihomo-γ-linolenic acid (DGLA) and arachidonic acid (AA) via desaturation and elongation pathways [42]. Differences in C18:3n-6 levels may result from genetic polymorphisms in *FASN* and *SCD*, which regulate fatty acid synthesis and desaturation [43]. In terms of meat quality, n-6 PUFAs, including C18:3n-6, affect marbling, tenderness, and oxidative stability. While higher PUFA levels enhance tenderness and juiciness, they also increase lipid oxidation, impacting shelf life and flavor stability [44,45]. In Hanwoo cattle, optimizing PUFA content and oxidative resistance is crucial for meat palatability and consumer acceptance. Although further research is needed, C18:3n-6 may contribute to improved beef flavor and nutritional value, supporting its relevance in selective breeding strategies.

Though the *PLAG1* gene primarily associated with growth traits and carcass characteristics, its indirect influence on fatty acid profile is also noteworthy [16,46]. *PLAG1* polymorphism showed a relation with palmitoleic acid and Eicosenoic acid in Japanese Black cattle and Hanwoo cattle, respectively [47,48]. Specific genotypes of *PLAG1* might affect the distribution and amount of intramuscular fat, subsequently altering beef’s SFA and MUFA content.

This study underscores the potential of SNP-based genetic selection in optimizing the fatty acid composition of beef. However, it also highlights the complexity of genetic and environmental interactions in determining beef quality. Future research should, therefore, extend beyond genetic factors to encompass external influences, such as diet and farming practices, to holistically understand and improve beef quality.

### 4.3. Relative Expression of Adipogenic Genes of Different Genotypes in Hanwoo Steer

This study provides valuable perception on the influence of SNP genotypes in *FASN*, *SCD*, and *PLAG1* genes on adipogenic gene expression and their consequent impact on lipid metabolism in beef cattle. The observed variations in gene expression patterns among these genotypes are crucial for understanding how genetic factors contribute to beef quality, particularly fat content and lipid composition.

The significant differences in *AMPKα* and *CPT1* expression among *FASN* genotypes suggest a genotype-dependent regulation of lipid metabolism pathways. Given that *AMPKα* inhibits fatty acid synthesis and promotes β-oxidation, its lower expression in *FASN*-AG genotypes may facilitate greater lipid accumulation, contributing to higher intramuscular fat (IMF) levels [49]. *CPT1* promotes the β-oxidation of fatty acids by catalyzing the formation of long-chain acylcarnitines and is an important mediator of fatty acid oxidation (FAO) [50]. The lower expression levels of *AMPKα* and *CPT1* in the *FASN*-AG genotype compared to AA and the higher levels in GG compared to AG types suggest a genotype-dependent modulation of fatty acid metabolism pathways. Previous studies in beef cattle have shown a negative correlation between *AMPKα* expression and IMF content, reinforcing the role of *AMPKα* in fat metabolism regulation [51]. *GPR43*, a short-chain fatty acid receptor, also exhibited expression variation across different genotypes. The observed trend shows that genotypes containing G allele demonstrate higher *GPR43* expression. The link between *GPR43* expression and intramuscular fat deposition remains underexplored in cattle, but studies in other livestock species indicate that *GPR43* activation enhances adipogenic pathways [52]. This suggests that *GPR43* corresponds with the established role of short-chain fatty acids in energy metabolism and adipogenesis [53]. *SCD* expression was highest in the GG genotype, supporting its role as a key regulator of monounsaturated fatty acid (MUFA) synthesis. *SCD* converts saturated fatty acids (C18:0) into MUFAs (C18:1), which are crucial for marbling, tenderness, and oxidative stability [32]. The association between *SCD* polymorphisms and increased MUFA content has been reported in multiple cattle breeds, further reinforcing *SCD*’s role as a genetic marker for meat quality improvement [54]. Such variations might influence the fatty acid profile and energy utilization in muscle tissues.

A key finding of this study is the significant variation in *SCD* gene expression across different genotypes, particularly its highest expression in *SCD*-GG genotypes. *SCD* catalyzes the conversion of saturated fatty acids (SFAs) into monounsaturated fatty acids (MUFAs), such as oleic acid (C18:1). Elevated *SCD* expression directly increases the proportion of MUFAs, which are associated with enhanced marbling, improved tenderness, and better oxidative stability in beef [54]. The significant variation in *GPR43* and *SCD* expression in T-containing genotypes further supports the idea that *SCD* polymorphisms influence lipid metabolism by altering the fatty acid profile. The correlation between higher *SCD* expression and increased unsaturated fatty acid content has been well documented in cattle [55]. Given that C18:1 is a major determinant of beef flavor and consumer preference, the association between *SCD* genotype and increased MUFA content highlights its potential use as a genetic marker for selective breeding programs aimed at improving meat quality. Furthermore, while the differences in *AMPKα* and *CPT1* expression between *SCD* CC and CT genotypes were not statistically significant, their modulation suggests a potential link between lipid oxidation and fatty acid synthesis in muscle tissues, which warrants further investigation.

Studies in Nellore cattle have identified *PLAG1* polymorphisms as significant contributors to carcass weight and fat deposition, highlighting its potential impact on IMF content and beef quality [18]. Compared to CC types, the *PLAG1* CG and GG genotypes showed higher expression levels of *AMPKα*, *CPT1*, and *SCD*, linking the *PLAG1* genotype to lipid metabolism [34,47]. *PLAG1* functions as a transcription factor that regulates genes involved in growth, metabolism, and adipogenesis. One of its key mechanisms is through the insulin-like growth factor (IGF) signaling pathway, where it upregulates IGF-2, leading to *IGF-1* receptor activation [56]. This pathway has been implicated in modulating *AMPKα* activity, which in turn regulates fatty acid metabolism by inhibiting lipogenesis and promoting fatty acid oxidation [57]. The higher *AMPKα* expression in *PLAG1* GG genotypes suggests that *PLAG1* may influence lipid metabolism by altering energy balance and adipogenic signaling. Since *AMPKα* suppresses fatty acid synthesis while enhancing β-oxidation, its increased expression in these genotypes could contribute to variations in fat deposition and beef quality traits. Similarly, the upregulation of *CPT1* and *SCD* in *PLAG1* CG and GG genotypes further supports the role of *PLAG1* in lipid mobilization and fatty acid desaturation, processes essential for intramuscular fat composition and marbling development in beef cattle.

This study’s findings reveal a complex interaction between genetic factors and adipogenic gene expression in beef cattle. The variations in expression levels of *AMPKα*, *CPT1*, *GPR43*, and *SCD* across different SNP genotypes underscore the potential of using genetic information to predict and influence fat composition and metabolism in beef production. However, it is crucial to integrate these genetic influences with environmental and dietary factors for a comprehensive understanding of beef quality traits. Future research should combine genetic, nutritional, and management strategies to optimize beef quality and cater to consumer preferences. Additionally, the identification of favorable SNPs in *FASN*, *SCD*, and *PLAG1* can serve as a basis for marker-assisted selection (MAS) and crossbreeding strategies in Hanwoo cattle. Incorporating these genetic markers into breeding programs could enhance traceability and quality control in beef production, supporting precision livestock farming.

## 5. Conclusions

This study investigated the effects of *FASN* g.16024 (A>G), *SCD* g.10329 (C>T), and *PLAG1* g.25003338 (C>G) SNPs on carcass traits and fatty acid composition in Hanwoo cattle. The primary findings indicate that specific genotypes (*FASN*-AG, *SCD*-CT, and *PLAG1*-GG) were more prevalent in the population and were positively associated with superior carcass quality and yield grades. These SNPs significantly influenced the fatty acid profile, affecting both saturated and unsaturated fatty acids, which suggests their potential role in lipid metabolism and beef quality improvement. Additionally, variations in the expression of adipogenic genes (*AMPKα*, *CPT1*, *GPR43*, and *SCD*) were observed among different genotypes, indicating a possible genetic influence on lipid metabolism and adipogenesis. These findings offer valuable insights into the genetic regulation of lipid metabolism and beef quality, emphasizing the potential of *FASN*, *SCD*, and *PLAG1* SNPs as selective breeding markers. Integrating these genetic variations into breeding programs could enhance intramuscular fat composition, marbling, and overall meat quality, contributing to the optimization of Hanwoo beef production. However, while these results advance our understanding of the molecular mechanisms of fat deposition, further functional validation is necessary to confirm their biological significance. However, the small sample size (n = 128) and uncontrolled environmental factors may limit the results’ generalizability. Despite these constraints, this study provides valuable insights for genetic selection and beef quality improvement in Hanwoo cattle.

## Figures and Tables

**Table 1:** Primer sequences of *FASN* g. 16024 G>A, *SCD* g. 10329 C>T, and *PLAG1* g. 25003338 C>G.

Gene	Primer Sequence (5′ to 3′)	Size (bp) and Amplified Region	Annealing Temp	Restriction Enzyme
*FASN* NC_037346.1	F: CTACCAAGCCAGGCAGGTC	337 (Exon 34)	52 °C	HhaI
	R: GCCATTGTACTTGGGCTTGT			
*SCD* NC_037353.1	F: TCAGGTAGGTCTCAGCGTC	494 (Exon 5)	52 °C	FauI
	R: CATTGTCATTTTTCACCCTTT			
*PLAG1* NC_037341.1	F: CAA GGG CTC AAC GTA GG	374 (3′UTR)	53 °C	HphI
	R: TGTTTCAAGTGCCATTAGAGG			

**Table 2:** Forward and reverse primers for real-time quantitative PCR for specific gene mRNA.

Gene	Gene ID	Sequence (5′ to 3′)
*RPS9*	DT860044	F: GAGCTGGGTTTGTCGCAAAA
		R: GGTCGAGGCGGGACTTCT
*AMPK-α*	NM_001109802	F: ACCATTCTTGGTTGCTGAAACTC
		R: CACCTTGGTGTTTGGATTTCTG
*CPT1β*	NM_001034349	F: ACACATCTACCTGTCCGTGATCA
		R: CCCCTGAGGATGCCATTCT
*GPR43*	FJ_562212	F: GGCTTTCCCCGTGCAGTA
		R: ATCAGAGCAGCGATCACTCCAT
*SCD*	AB075020	F: TGCCCACCACAAGTTTTCAG
		R: GCCAACCCACGTGAGAGAAG

**Table 3:** Genotype frequencies and allele frequencies with carcass traits in Hanwoo steer.

	*FASN* g. 16024 G>A					χ^2^	*SCD* g. 10329 C>T					χ^2^	*PLAG1* g. 25003338 C>G					χ^2^
	Genotype			Allele			Genotype			Allele			Genotype			Allele		
	AA (n = 22)	AG (n = 45)	GG (n = 41)	A	G		CC (n = 12)	CT (n = 54)	TT (n = 33)	C	T		CC (n = 9)	CG (n = 39)	GG (n = 51)	C	G	
	0.204	0.417	0.380	0.412	0.588	0.347	0.121	0.545	0.333	0.394	0.606	0.367	0.091	0.394	0.515	0.288	0.712	0.927
Quality Grade																		
1++	0.102	0.176	0.130	0.190	0.218		0.040	0.232	0.131	0.157	0.247		0.020	0.162	0.232	0.101	0.313	
1+	0.065	0.120	0.167	0.125	0.227		0.040	0.192	0.131	0.136	0.227		0.051	0.162	0.172	0.131	0.253	
1	0.028	0.083	0.074	0.069	0.116		0.040	0.091	0.051	0.086	0.096		0.000	0.061	0.091	0.030	0.121	
2	0.009	0.037	0.009	0.028	0.028		0.000	0.030	0.020	0.015	0.035		0.020	0.010	0.020	0.025	0.025	
Yield Grade																		
A	0.056	0.065	0.065	0.088	0.097		0.030	0.101	0.091	0.081	0.141		0.051	0.071	0.091	0.086	0.126	
B	0.120	0.269	0.231	0.255	0.366		0.061	0.323	0.192	0.222	0.354		0.040	0.242	0.323	0.162	0.444	
C	0.028	0.083	0.083	0.069	0.125		0.030	0.121	0.051	0.091	0.111		0.000	0.081	0.101	0.040	0.141	

Yield index formula for steers: [11.06398 − 1.25149 × Backfat thickness (mm) + 0.28293 × Ribeye area (cm^2^) + 0.54768 × Carcass cold weight (kg)] ÷ Carcass cold weight (kg) × 100. A > 62.52, 62.52 > B > 60.40, C > 60.40.

**Table 4:** Muscle tissue and adipose tissue fatty acid composition (mg/100 mL) of *FASN* g. 16024 G>A in Hanwoo steer.

	Muscle Tissue			*p*-Value	Adipose Tissue			*p*-Value
	AA	AG	GG		AA	AG	GG	
Decanoic acid (C10:0)	4.35 ± 2.39	3.58 ± 3.16	2.66 ± 3.42	0.22	0.62 ± 1.86	1.47 ± 2.46	0.85 ± 2.03	0.564
Lauric acid (C12:0)	0.07 ± 0.03	0.06 ± 0.04	0.06 ± 0.04	0.354	0.12 ± 0.03 ^ab^	0.13 ± 0.03 ^a^	0.10 ± 0.03 ^b^	0.018
Myristic acid (C14:0)	2.27 ± 0.32	2.40 ± 0.56	2.20 ± 0.50	0.302	3.11 ± 0.32	3.66 ± 0.64	3.63 ± 0.93	0.163
Myristoleic acid (C14:1)	0.61 ± 0.23	0.66 ± 0.27	0.57 ± 0.24	0.354	2.36 ± 0.54	2.20 ± 0.69	2.40 ± 1.07	0.765
Palmitic acid (C16:0)	26.07 ± 1.62	26.74 ± 1.99	27.01 ± 1.44	0.225	23.36 ± 1.27	24.32 ± 2.11	24.49 ± 1.87	0.323
Palmitoleic acid (C16:1)	4.08 ± 0.58	4.15 ± 0.89	3.96 ± 0.75	0.653	7.79 ± 1.53	7.74 ± 1.37	8.45 ± 1.67	0.330
Stearic acid (C18:0)	9.37 ± 1.19	9.76 ± 1.24	9.89 ± 1.29	0.427	5.98 ± 1.55	6.68 ± 1.49	6.30 ± 1.19	0.438
Oleic acid (C18:1)	45.55 ± 1.87	44.69 ± 2.72	45.16 ± 2.99	0.595	50.21 ± 1.66	47.79 ± 3.95	47.86 ± 3.79	0.208
Linoleic (C18:2n-6)	3.86 ± 1.22	4.02 ± 1.89	4.40 ± 1.40	0.483	2.68 ± 0.34	2.55 ± 0.41	2.59 ± 0.47	0.771
γ-Linoleic (C18:3n-6)	0.09 ± 0.04 ^a^	0.08 ± 0.07 ^a^	0.04 ± 0.05 ^b^	0.013	0.21 ± 0.04	0.19 ± 0.03	0.19 ± 0.03	0.151
Linolenic (C18:3n-3)	0.13 ± 0.02	0.11 ± 0.07	0.22 ± 0.51	0.482	0.16 ± 0.02	0.15 ± 0.03	0.15 ± 0.03	0.530
Arachidic acid (C20:0)	0.03 ± 0.03	0.03 ± 0.03	0.02 ± 0.03	0.249	0.04 ± 0.04	0.05 ± 0.04	0.03 ± 0.04	0.466
Heneicosanoic acid (C20:1)	0.30 ± 0.08	0.25 ± 0.1	0.26 ± 0.09	0.34	0.53 ± 0.15 ^a^	0.42 ± 0.14 ^b^	0.39 ± 0.13 ^b^	0.037
Eicosatrienoic acid (C20:2n-6)	0.02 ± 0.05	0.01 ± 0.03	0.02 ± 0.04	0.618	0.02 ± 0.04 ^a^	0.00 ± 0.00 ^b^	0.00 ± 0.02 ^ab^	0.079
C20:3n-6	0.48 ± 0.26	0.56 ± 0.36	0.60 ± 0.25	0.416	0.20 ± 0.06	0.21 ± 0.04	0.16 ± 0.07	0.063
C20:4n-6	0.96 ± 0.58	1.09 ± 0.84	1.22 ± 0.61	0.493	0.06 ± 0.06	0.06 ± 0.05	0.04 ± 0.05	0.428
Total SFAs ^(1)^	43.03 ± 2.96	43.50 ± 3.61	42.70 ± 3.31	0.669	34.21 ± 2.20 ^b^	37.27 ± 4.37 ^a^	36.35 ± 3.38 ^ab^	0.131
Total MUFAs ^(2)^	51.19 ± 2.24	50.40 ± 2.90	50.61 ± 2.74	0.656	61.76 ± 1.99	58.92 ± 4.39	59.87 ± 3.16	0.158
Total PUFAs ^(3)^	5.55 ± 2.00	5.87 ± 2.98	6.50 ± 2.19	0.426	3.34 ± 0.46	3.15 ± 0.45	3.14 ± 0.52	0.565
UFAs/SFAs ^(4)^	1.33 ± 0.16	1.31 ± 0.19	1.35 ± 0.18	0.661	1.91 ± 0.18 ^a^	1.70 ± 0.30 ^b^	1.76 ± 0.25 ^ab^	0.147
ω3/ω6	0.03 ± 0.01	0.02 ± 0.02	0.03 ± 0.07	0.695	0.05 ± 0.01	0.05 ± 0.01	0.05 ± 0.01	0.488

(1) SFAs: saturated fatty acids. (2) MUFAs: monounsaturated fatty acids. (3) PUFAs: polyunsaturated fatty acids. (4) UFAs: unsaturated fatty acids. a, b: Mean in the same line with different superscripts significantly differed (*p* < 0.05).

**Table 5:** Muscle tissue and adipose tissue fatty acid composition (mg/100 mL) of *SCD* g. 10329 (C>T) in Hanwoo steer.

	Muscle Tissue			*p*-Value	Adipose Tissue			*p*-Value
	CC	CT	TT		CC	CT	TT	
Decanoic acid (C10:0)	2.25 ± 3.12	3.05 ± 3.12	4.05 ± 3.51	0.363	0.8 ± 2.11	1 ± 2.13	1.3 ± 2.37	0.882
Lauric acid (C12:0)	0.06 ± 0.03	0.06 ± 0.05	0.06 ± 0.05	0.97	0.11 ± 0.03	0.12 ± 0.03	0.11 ± 0.04	0.726
Myristic acid (C14:0)	2.09 ± 0.27	2.26 ± 0.53	2.33 ± 0.53	0.53	3.12 ± 0.66	3.58 ± 0.67	3.65 ± 0.85	0.279
Myristoleic acid (C14:1)	0.77 ± 0.18 ^a^	0.65 ± 0.32 ^ab^	0.51 ± 0.27 ^b^	0.086	2.61 ± 0.9 ^a^	2.56 ± 0.9 ^a^	1.85 ± 0.56 ^b^	0.047
Palmitic acid (C16:0)	26.59 ± 1.45	26.74 ± 1.57	26.42 ± 1.68	0.768	23.54 ± 1.76	24.33 ± 1.52	24.01 ± 1.94	0.562
Palmitoleic acid (C16:1)	3.9 ± 0.44	4.1 ± 0.99	4.12 ± 0.83	0.834	6.89 ± 1.67 ^b^	8.58 ± 1.33 ^a^	8.07 ± 1.02 ^a^	0.019
Stearic acid (C18:0)	9.15 ± 0.78	9.62 ± 1.39	10.11 ± 1.52	0.217	6.69 ± 1.61	5.88 ± 0.9	6.43 ± 0.87	0.14
Oleic acid (C18:1)	46.4 ± 2.7	45.26 ± 2.81	44.66 ± 2.59	0.324	49.78 ± 2.92	48 ± 3.36	48.68 ± 4.16	0.513
Linoleic (C18:2n-6)	4.46 ± 1.62	4.26 ± 1.58	3.95 ± 1.67	0.703	2.75 ± 0.44	2.57 ± 0.41	2.57 ± 0.51	0.629
γ-Linoleic (C18:3n-6)	0.07 ± 0.06	0.07 ± 0.07	0.08 ± 0.05	0.869	0.22 ± 0.03 ^a^	0.2 ± 0.03 ^ab^	0.18 ± 0.03 ^b^	0.041
Linolenic (C18:3n-3)	0.15 ± 0.04	0.2 ± 0.47	0.1 ± 0.06	0.627	0.15 ± 0.03	0.15 ± 0.03	0.14 ± 0.02	0.432
Arachidic acid (C20:0)	0.02 ± 0.03	0.02 ± 0.03	0.03 ± 0.03	0.564	0.04 ± 0.04	0.03 ± 0.03	0.05 ± 0.04	0.195
Heneicosanoic acid (C20:1)	0.3 ± 0.08	0.27 ± 0.09	0.24 ± 0.09	0.282	0.48 ± 0.14	0.41 ± 0.13	0.44 ± 0.16	0.555
Eicosatrienoic acid (C20:2n-6)	0 ± 0	0.02 ± 0.04	0.03 ± 0.04	0.234	0.01 ± 0.03	0 ± 0	0.01 ± 0.03	0.28
C20:3n-6	0.65 ± 0.3	0.58 ± 0.28	0.5 ± 0.34	0.471	0.2 ± 0.03	0.19 ± 0.07	0.18 ± 0.07	0.839
C20:4n-6	1.29 ± 0.64	1.17 ± 0.69	1 ± 0.82	0.566	0.04 ± 0.06	0.05 ± 0.05	0.06 ± 0.05	0.648
Total SFAs ^(1)^	41.07 ± 2.17 ^b^	42.59 ± 3.36 ^ab^	43.98 ± 2.95 ^a^	0.077	35.39 ± 2.56	35.85 ± 3.11	36.44 ± 3.57	0.77
Total MUFAs ^(2)^	52.08 ± 2.51	50.91 ± 2.7	50.17 ± 2.51	0.225	60.6 ± 2.82	60.32 ± 2.95	59.82 ± 3.44	0.849
Total PUFAs ^(3)^	6.62 ± 2.48	6.29 ± 2.52	5.66 ± 2.69	0.587	3.36 ± 0.48	3.16 ± 0.48	3.13 ± 0.55	0.585
UFAs/SFAs ^(4)^	1.44 ± 0.13 ^a^	1.36 ± 0.18 ^ab^	1.28 ± 0.15 ^b^	0.075	1.82 ± 0.18	1.79 ± 0.23	1.75 ± 0.27	0.831
ω3/ω6	0.03 ± 0.01	0.03 ± 0.07	0.02 ± 0.01	0.723	0.05 ± 0	0.05 ± 0.01	0.05 ± 0	0.269

(1) SFAs: saturated fatty acids. (2) MUFAs: monounsaturated fatty acids. (3) PUFAs: polyunsaturated fatty acids. (4) UFAs: unsaturated fatty acids. a, b: Mean in the same line with different superscripts significantly differed (*p* < 0.05).

**Table 6:** Muscle tissue and adipose tissue fatty acid composition (mg/100 mL) of *PLAG1* g. 25003338 (C>G) in Hanwoo steer.

	Muscle Tissue			*p*-Value	Adipose Tissue			*p*-Value
	CC	CG	GG		CC	CG	GG	
Decanoic acid (C10:0)	4.9 ± 2.83	2.71 ± 3.31	3.31 ± 3.14	0.351	2.13 ± 2.95	1.09 ± 2.17	0.93 ± 2.13	0.553
Lauric acid (C12:0)	0.09 ± 0.02	0.06 ± 0.04	0.06 ± 0.04	0.202	0.16 ± 0.03 ^a^	0.12 ± 0.03 ^b^	0.1 ± 0.02 ^b^	0.001
Myristic acid (C14:0)	2.71 ± 0.64 ^a^	2.33 ± 0.54 ^ab^	2.14 ± 0.42 ^b^	0.04	4.47 ± 0.88 ^a^	3.6 ± 0.89 ^b^	3.37 ± 0.5 ^b^	0.01
Myristoleic acid (C14:1)	0.75 ± 0.24	0.65 ± 0.27	0.55 ± 0.24	0.118	2.47 ± 1.13	2.08 ± 0.93	2.4 ± 0.72	0.483
Palmitic acid (C16:0)	25.71 ± 2.67	26.75 ± 1.84	26.7 ± 1.39	0.441	25.74 ± 2.64	24.31 ± 2.28	24.03 ± 1.29	0.174
Palmitoleic acid (C16:1)	4.66 ± 1.04 ^a^	4.13 ± 0.84 ^ab^	3.9 ± 0.74 ^b^	0.121	7.81 ± 1.86	7.58 ± 1.54	8.33 ± 1.2	0.267
Stearic acid (C18:0)	9.48 ± 1.28	9.9 ± 1.3	9.76 ± 1.3	0.776	7.85 ± 1.71 ^a^	6.68 ± 1.16 ^b^	5.91 ± 0.88 ^b^	0.002
Oleic acid (C18:1)	44.5 ± 3.24	44.94 ± 3.17	45.57 ± 2.29	0.556	43.47 ± 4.32 ^b^	48.63 ± 4.01 ^a^	48.86 ± 2.61 ^a^	0.007
Linoleic (C18:2n-6)	3.54 ± 1.27	4.46 ± 1.81	4.12 ± 1.44	0.444	2.57 ± 0.19	2.52 ± 0.46	2.65 ± 0.45	0.674
γ-Linoleic (C18:3n-6)	0.07 ± 0.07	0.07 ± 0.07	0.06 ± 0.05	0.753	0.17 ± 0.05 ^b^	0.2 ± 0.03 ^a^	0.19 ± 0.03 ^ab^	0.153
Linolenic (C18:3n-3)	0.16 ± 0.04	0.1 ± 0.07	0.21 ± 0.48	0.47	0.16 ± 0.03	0.15 ± 0.03	0.15 ± 0.02	0.454
Arachidic acid (C20:0)	0.03 ± 0.03	0.02 ± 0.03	0.02 ± 0.03	0.766	0.07 ± 0.01 ^a^	0.04 ± 0.04 ^b^	0.03 ± 0.03 ^b^	0.072
Heneicosanoic acid (C20:1)	0.27 ± 0.11	0.23 ± 0.13	0.28 ± 0.06	0.148	0.3 ± 0.16 ^b^	0.46 ± 0.16 ^a^	0.42 ± 0.12 ^ab^	0.087
Eicosatrienoic acid (C20:2n-6)	0.01 ± 0.02	0.02 ± 0.04	0.02 ± 0.04	0.837	0 ± 0	0.01 ± 0.02	0 ± 0.02	0.809
C20:3n-6	0.44 ± 0.16	0.59 ± 0.34	0.56 ± 0.28	0.6	0.18 ± 0.11	0.19 ± 0.07	0.19 ± 0.04	0.925
C20:4n-6	0.83 ± 0.44	1.21 ± 0.82	1.1 ± 0.65	0.533	0.05 ± 0.06	0.04 ± 0.05	0.05 ± 0.05	0.723
Total SFAs ^(1)^	43.8 ± 2.88	42.74 ± 3.44	42.81 ± 3.38	0.806	41.49 ± 4.61 ^a^	36.82 ± 3.13 ^b^	35.28 ± 2.61 ^b^	0.001
Total MUFAs ^(2)^	50.85 ± 3.17	50.63 ± 3.03	50.92 ± 2.42	0.916	54.78 ± 4.58 ^b^	59.52 ± 3.09 ^a^	60.81 ± 2.57 ^a^	0.001
Total PUFAs ^(3)^	5.05 ± 1.8	6.44 ± 2.84	6.06 ± 2.37	0.517	3.12 ± 0.29	3.1 ± 0.52	3.23 ± 0.49	0.698
UFAs/SFAs ^(4)^	1.29 ± 0.16	1.35 ± 0.18	1.35 ± 0.18	0.764	1.42 ± 0.29 ^b^	1.72 ± 0.23 ^a^	1.83 ± 0.2 ^a^	0.002
ω3/ω6	0.03 ± 0.01	0.02 ± 0.01	0.04 ± 0.07	0.41	0.05 ± 0.01	0.05 ± 0.01	0.05 ± 0.01	0.323

(1) SFAs: saturated fatty acids. (2) MUFAs: monounsaturated fatty acids. (3) PUFAs: polyunsaturated fatty acids. (4) UFAs: unsaturated fatty acids. a, b: Mean in the same line with different superscripts significantly differed (*p* < 0.05).

**Table 7:** Effects of *FASN* g. 16024 G>A genotype and quality grade on fatty acid composition in Hanwoo steer.

	Muscle Tissue			Adipose Tissue		
	Genotype (G)	Quality Grade (Q)	G×Q	Genotype (G)	Quality Grade (Q)	G×Q
Decanoic acid (C10:0)	N.S.	N.S.	N.S.	N.S.	*	N.S.
Lauric acid (C12:0)	N.S.	N.S.	N.S.	N.S.	N.S.	N.S.
Myristic acid (C14:0)	N.S.	N.S.	N.S.	N.S.	N.S.	N.S.
Myristoleic acid (C14:1)	N.S.	N.S.	N.S.	N.S.	N.S.	N.S.
Palmitic acid (C16:0)	N.S.	N.S.	N.S.	N.S.	N.S.	N.S.
Palmitoleic acid (C16:1)	N.S.	N.S.	N.S.	N.S.	N.S.	N.S.
Stearic acid (C18:0)	N.S.	N.S.	*	N.S.	N.S.	N.S.
Oleic acid (C18:1)	N.S.	N.S.	N.S.	N.S.	N.S.	N.S.
Linoleic (C18:2n-6)	N.S.	*	N.S.	N.S.	N.S.	N.S.
γ-Linoleic (C18:3n-6)	*	*	N.S.	N.S.	N.S.	N.S.
Linolenic (C18:3n-3)	N.S.	N.S.	N.S.	N.S.	N.S.	N.S.
Arachidic acid (C20:0)	N.S.	N.S.	N.S.	N.S.	N.S.	N.S.
Heneicosanoic acid (C20:1)	*	N.S.	N.S.	N.S.	N.S.	N.S.
Eicosatrienoic acid (C20:2n-6)	N.S.	N.S.	N.S.	*	*	*
C20:3n-6	N.S.	N.S.	N.S.	N.S.	N.S.	N.S.
C20:4n-6	N.S.	*	N.S.	N.S.	N.S.	N.S.
Total SFAs ^(1)^	N.S.	*	N.S.	N.S.	N.S.	N.S.
Total MUFAs ^(2)^	N.S.	N.S.	N.S.	N.S.	N.S.	N.S.
Total PUFAs ^(3)^	N.S.	*	N.S.	N.S.	N.S.	N.S.
UFAs/SFAs ^(4)^	N.S.	*	N.S.	N.S.	N.S.	N.S.
ω3/ω6	N.S.	N.S.	N.S.	*	N.S.	N.S.

(1) SFAs: saturated fatty acids. (2) MUFAs: monounsaturated fatty acids. (3) PUFAs: polyunsaturated fatty acids. (4) UFAs: unsaturated fatty acids. N.S.: Non-significant. * *p* < 0.05.

**Table 8:** Effects of *SCD* g. 10329 C>T genotype and quality grade on fatty acid composition in Hanwoo steer.

	Muscle Tissue			Adipose Tissue		
	Genotype (G)	Quality Grade (Q)	G×Q	Genotype (G)	Quality Grade (Q)	G×Q
Decanoic acid (C10:0)	N.S.	N.S.	N.S.	N.S.	N.S.	N.S.
Lauric acid (C12:0)	N.S.	N.S.	N.S.	N.S.	N.S.	N.S.
Myristic acid (C14:0)	N.S.	N.S.	N.S.	N.S.	N.S.	N.S.
Myristoleic acid (C14:1)	N.S.	N.S.	N.S.	*	N.S.	N.S.
Palmitic acid (C16:0)	N.S.	N.S.	N.S.	N.S.	N.S.	N.S.
Palmitoleic acid (C16:1)	N.S.	N.S.	N.S.	*	N.S.	N.S.
Stearic acid (C18:0)	N.S.	N.S.	N.S.	N.S.	N.S.	N.S.
Oleic acid (C18:1)	N.S.	*	N.S.	N.S.	N.S.	N.S.
Linoleic (C18:2n-6)	N.S.	*	N.S.	N.S.	N.S.	N.S.
γ-Linoleic (C18:3n-6)	N.S.	N.S.	N.S.	*	N.S.	N.S.
Linolenic (C18:3n-3)	N.S.	N.S.	N.S.	N.S.	N.S.	N.S.
Arachidic acid (C20:0)	N.S.	N.S.	N.S.	N.S.	N.S.	N.S.
Heneicosanoic acid (C20:1)	*	N.S.	*	N.S.	N.S.	N.S.
Eicosatrienoic acid (C20:2n-6)	N.S.	N.S.	N.S.	N.S.	***	N.S.
C20:3n-6	N.S.	N.S.	N.S.	N.S.	N.S.	N.S.
C20:4n-6	N.S.	N.S.	N.S.	N.S.	N.S.	N.S.
Total SFAs ^(1)^	N.S.	N.S.	N.S.	N.S.	N.S.	N.S.
Total MUFAs ^(2)^	N.S.	*	N.S.	N.S.	N.S.	N.S.
Total PUFAs ^(3)^	N.S.	*	N.S.	N.S.	N.S.	N.S.
UFAs/SFAs ^(4)^	N.S.	N.S.	N.S.	N.S.	N.S.	N.S.
ω3/ω6	N.S.	N.S.	N.S.	N.S.	N.S.	N.S.

(1) SFAs: saturated fatty acids. (2) MUFAs: monounsaturated fatty acids. (3) PUFAs: polyunsaturated fatty acids. (4) UFAs: unsaturated fatty acids. N.S.: Non-significant. * *p* < 0.05. *** *p* < 0.0001.

**Table 9:** Effects of *PLAG1* g. 25003338 C>G genotype and quality grade on fatty acid composition in Hanwoo steer.

	Muscle Tissue			Adipose Tissue		
	Genotype (G)	Quality Grade (Q)	G×Q	Genotype (G)	Quality Grade (Q)	G×Q
Decanoic acid (C10:0)	N.S.	*	N.S.	N.S.	*	N.S.
Lauric acid (C12:0)	*	N.S.	N.S.	*	N.S.	N.S.
Myristic acid (C14:0)	*	N.S.	N.S.	*	N.S.	N.S.
Myristoleic acid (C14:1)	*	N.S.	N.S.	N.S.	N.S.	N.S.
Palmitic acid (C16:0)	***	*	***	N.S.	N.S.	N.S.
Palmitoleic acid (C16:1)	**	*	*	N.S.	N.S.	N.S.
Stearic acid (C18:0)	N.S.	N.S.	N.S.	*	N.S.	N.S.
Oleic acid (C18:1)	***	N.S.	***	*	N.S.	N.S.
Linoleic (C18:2n-6)	N.S.	N.S.	*	N.S.	N.S.	N.S.
γ-Linoleic (C18:3n-6)	N.S.	N.S.	N.S.	N.S.	N.S.	N.S.
Linolenic (C18:3n-3)	N.S.	N.S.	N.S.	N.S.	N.S.	N.S.
Arachidic acid (C20:0)	N.S.	N.S.	N.S.	N.S.	N.S.	N.S.
Heneicosanoic acid (C20:1)	*	N.S.	N.S.	N.S.	N.S.	N.S.
Eicosatrienoic acid (C20:2n-6)	N.S.	N.S.	N.S.	N.S.	**	N.S.
C20:3n-6	N.S.	N.S.	N.S.	N.S.	N.S.	N.S.
C20:4n-6	N.S.	N.S.	N.S.	N.S.	N.S.	N.S.
Total SFAs ^(1)^	**	*	***	*	N.S.	N.S.
Total MUFAs ^(2)^	***	N.S.	***	*	N.S.	N.S.
Total PUFAs ^(3)^	N.S.	N.S.	N.S.	N.S.	N.S.	N.S.
UFAs/SFAs ^(4)^	***	***	***	*	N.S.	N.S.
ω3/ω6	N.S.	N.S.	N.S.	N.S.	N.S.	N.S.

(1) SFAs: saturated fatty acids. (2) MUFAs: monounsaturated fatty acids. (3) PUFAs: polyunsaturated fatty acids. (4) UFAs: unsaturated fatty acids. N.S.: Non-significant. * *p* < 0.05. ** *p* < 0.001. *** *p* < 0.0001.

## Data Availability

Data will be made available on reasonable request.

## Abbreviations

The following abbreviations are used in this manuscript:

SNP	Single Nucleotide Polymorphism
QTL	Quantitative Trait Locus
FASN	Fatty Acid Synthase
SCD	Stearoyl-CoA Desaturase
PLAG1	Pleomorphic Adenoma gene 1
RPS9	Ribosomal protein S9
AMPK-α	AMP-activated protein kinase-α
CPT1β	Carnitine Palmitoyl Transferase-1β
GPR43	G-coupled protein receptor-43
PCR-RFLP	Polymerase chain reaction–restriction fragment length polymorphism
PCR	Polymerase Chain Reaction
BF3	Boron trifluoride
KOH	Potassium hydroxide
FID	Fréchet inception distance
GLM	General Linear Model
HWE	Hardy–Weinberg Equilibrium
DMRT	Duncan’s Multiple Range test
SEM	Standard Error Mean
RT-qPCR	Real-time polymerase chain reaction
SFA	Saturated fatty acid
MUFA	Monounsaturated fatty acid
